# Bacterial transfer and biofilm formation in needleless connectors in a clinically simulated in vitro catheter model

**DOI:** 10.1017/ice.2023.60

**Published:** 2023-11

**Authors:** Marcia Ryder, Elinor deLancey-Pulcini, Albert E. Parker, Garth A. James

**Affiliations:** 1 Ryder Science, Brentwood, Tennessee; 2 Center for Biofilm Engineering, Montana State University, Bozeman, Montana; 3 Department of Mathematical Sciences, Montana State University, Bozeman, Montana

## Abstract

**Objective:**

Although needleless connectors (NCs) are widely used in clinical practice, they carry significant risk of bloodstream infection (BSI). In this study, we quantified differences in bacterial transfer and biofilm formation between various NCs.

**Design:**

Prospective, clinically simulated in vitro experimental study.

**Methods:**

We tested 20 NCs in a 5-day clinical simulation of *Staphylococcus aureus* inoculations onto NC septum surfaces, which were then flushed with saline and cultured for bacterial transfer. Biofilm formation was measured through destructive sampling of the connector-catheter system. Moreover, 8 NC design factors were evaluated for their influence on bacterial transfer and biofilm formation. This study was designed without a disinfection protocol to ascertain the intrinsic risk of each NC.

**Results:**

Clave Neutron and MicroClave had the lowest overall mean log density of bacteria in the flush compared to other NCs (*P* < .05), except there were no statistically significant differences between Clave Neutron, Microclave, SafeTouch, and SafeAccess (*P* ≥ .05). The amount of biofilm in the NC was positively associated with bacteria in the flush (*P <* .0005). Among 8 design factors, flow path was most important, with the internal cannula associated with a statistically significant 1 log reduction (LR) in bacteria in the flush (*R* ^2^ = 49%) and 0.5–2 LR in the connector (*R* ^2^ = 34%). All factors together best explained bacteria in the flush (*R* ^2^ = 65%) and biofilm in the connector (*R* ^2^ = 48%).

**Conclusions:**

Bacterial transfer and biofilm formation in the connector-catheter system varied statistically significantly between the 20 NCs, suggesting that NC choice can lower the risk of developing catheter-related BSIs.

Needleless connectors (NCs) were introduced in the 1990s to minimize risks of needlestick injuries, bloodborne pathogen exposure, and occupational hazards among healthcare workers. However, their use unexpectedly led to increased central-line–associated bloodstream infections (CLABSI) and thrombotic catheter occlusions.^1^ Several generations of device design enhancements evolved to avert these problems with variable results.

CLABSIs are serious healthcare-associated infections, accountable for 15 excess deaths per 100 events with an average cost of $48,108 per occurrence.^2^ Over the past decade, focused infection prevention programs reduced CLABSI rates in the United States. However, the COVID-19 pandemic thwarted this progress.^3^ Some hospitals reported as much as a 420% increase in CLABSI rates early in the pandemic compared to pre–COVID-19 occurrences.^4^ Recently, an analysis of National Healthcare Safety Network data showed a significant increase in standardized infection ratio to 1.04 in the third quarter of 2021 (2021-Q3), higher than any previous quarter since the prepandemic year 2019 (0.69, 2019-Q3).^5^ Factors contributing to this rise include increased patient volume and acuity, increased or prolonged vascular-access requirements, staffing and supply challenges, all of which resulted in substandard infection control practices, including time-critical tasks such as NC disinfection.^2,3,5–7^ Notably, inadequate disinfection increases the likelihood of bacterial transfer from septum surfaces into NCs, bacterial attachment within intraluminal flow paths, recalcitrant biofilm formation, and the development of catheter-related bloodstream infections (CRBSIs) through the flush.^8,9^

The 2022 SHEA/APIC/IDSA Society for Healthcare Epidemiology of America (SHEA)–Association for Professionals in Infection Control and Epidemiology (APIC)–Infectious Diseases Society of America (IDSA) practice recommendations^10^ and 2021 Intravenous Nursing Society Standards^11^ both acknowledged that although various device designs exist, optimal NC designs for infection prevention are unclear. NC design features vary, namely with complexities in mechanism of access, access portal, flow path, type of fluid displacement, and hydrodynamics (Fig. 1).^12^ The contribution of these factors to infection risk, however, remains unknown.

**Fig. 1.**
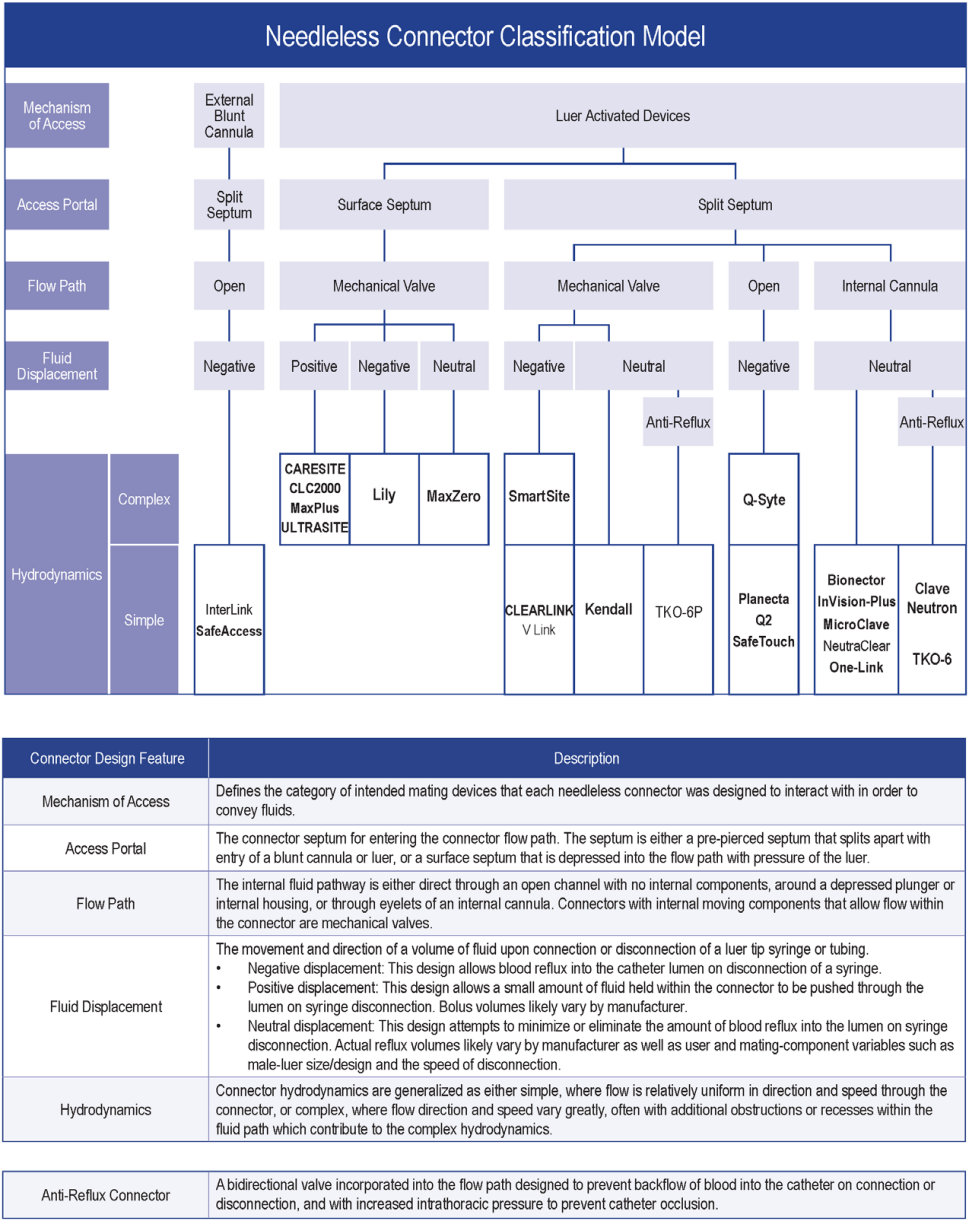
Classification model of needleless connector design for currently available devices. Device names in bold were evaluated as part of this study.

The number of marketed NCs has increased rapidly, with >30 designs available globally today. Varying design features pose significant challenges in selecting a NC with minimal infection risk. Here, 20 nondisinfected NCs were studied 15 of which are currently sold in the United States (Fig. 2). This prospective, clinically simulated in vitro study aimed to quantify differences in bacterial transfer through the NC as well as intraluminal biofilm formation in the NC, hub, and catheter lumen after repeated inoculations and flushing over a 5-day period. Bacteria transferred into the flush were a composite of bacteria inoculated onto the NC septum surface and detached from the biofilm, which clinically represents bloodstream entry (Fig. 3A). Moreover, 8 NC design factors were evaluated to determine their influence on bacterial transfer and biofilm formation.

**Fig. 2.**
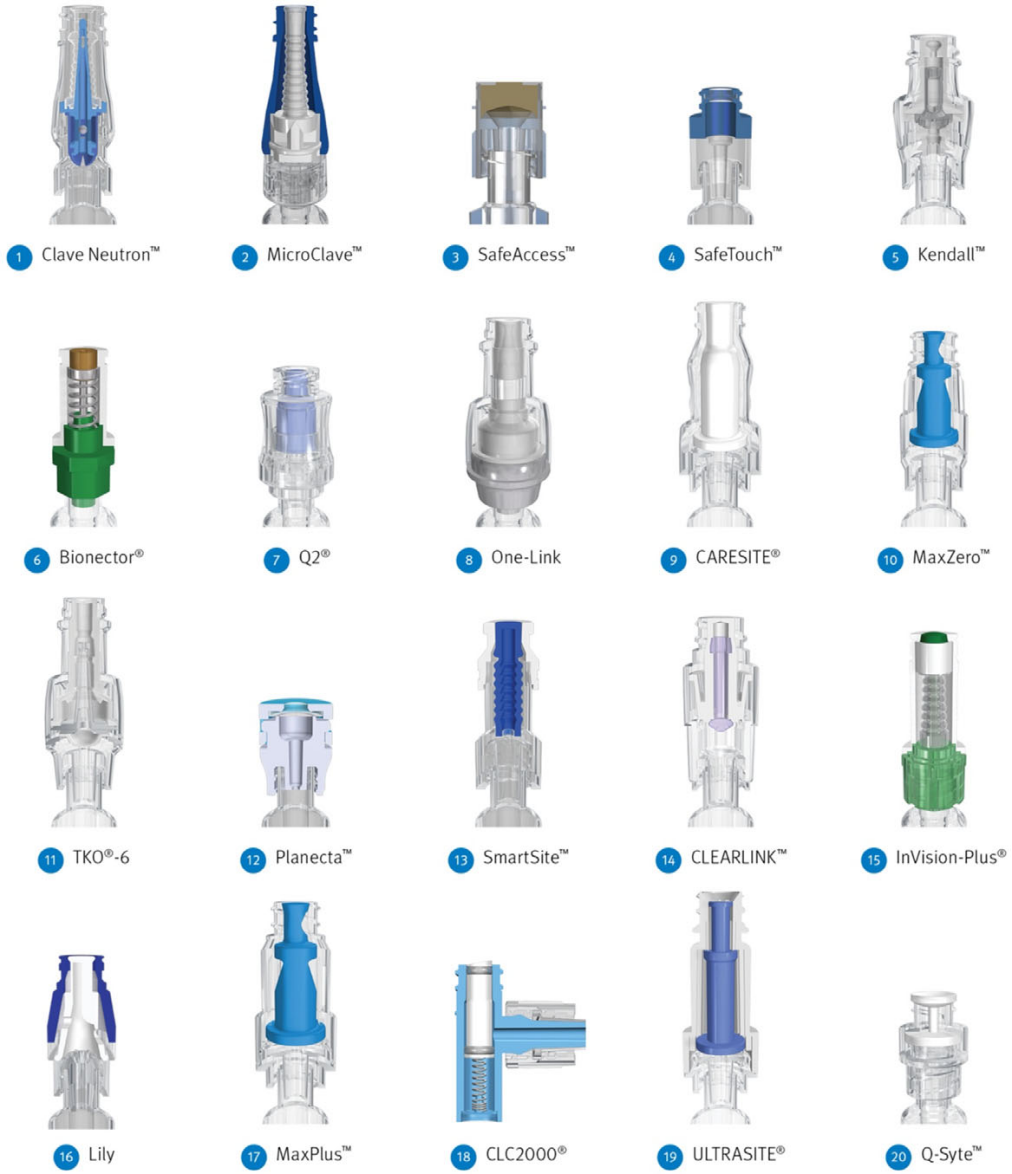
Cross-sectional view of each connector model arranged in order of the mean bacterial transfer over 5 days from the lowest to the highest number of colony-forming units. 1. Clave Neutron (ICU Medical, San Clemente, CA); 2. MicroClave (ICU Medical); 3. SafeAccess (Covidien, Dublin, Ireland); 4. SafeTouch (Nipro, Osaka, Japan); 5. Kendall (Covidien, Dublin, Ireland); 6. Bionector (Vygon SA, Ecouen, France); 7. Q2 (Quest Medical, Allen, TX); 8. One-Link (Baxter Healthcare, Deerfield, IL); 9. CARESITE (B. Braun Medical, Bethlehem, PA); 10. MaxZero (BD Medical, Franklin Lakes, NJ); 11. TKO-6 (Nexus Medical, Lenexa, KS); 12. Planecta (JMS, Hiroshima, Japan); 13. SmartSite (BD Medical); 14. CLEARLINK (Baxter Healthcare); 15. InVision-Plus (RyMed Technologies, Franklin, TN); 16. Lily (LILY Medical, Miaoli County, Taiwan); 17. MaxPlus (BD Medical); 18. CLC2000 (ICU Medical); 19. ULTRASITE (B. Braun Medical, Bethlehem, PA); 20. Q-Syte (BD Medical).

**Fig. 3.**
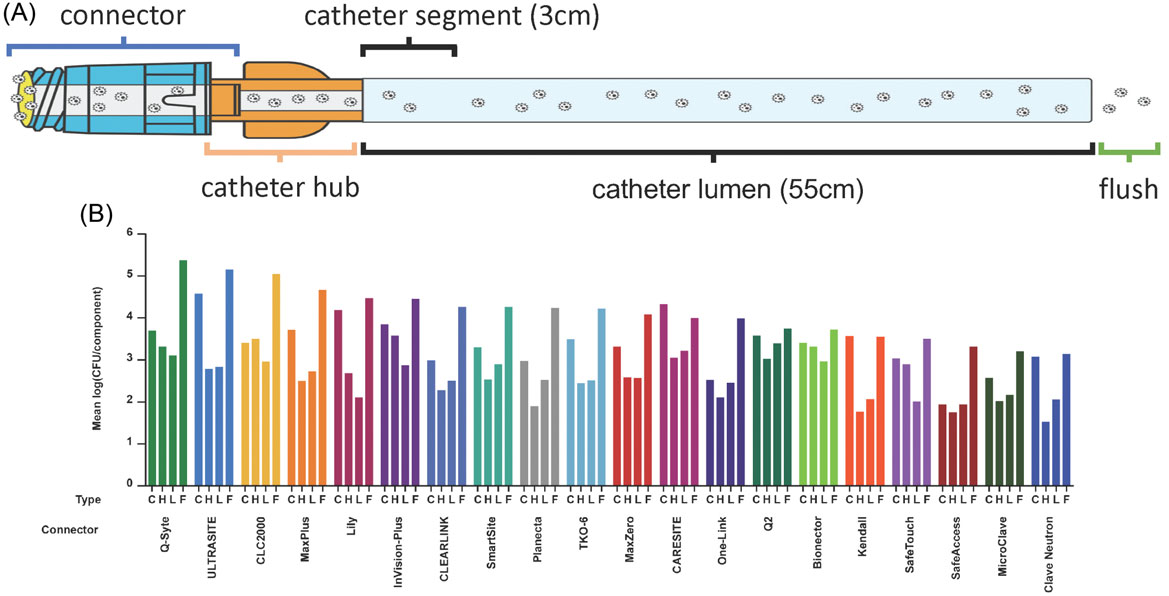
Four sampling components and their mean LDs of bacteria across connectors. (A) Four sampling components included the connector, catheter hub, catheter lumen, and flush. (B) The mean LDs of bacteria in each component are shown. Connectors are ordered from left to right from the highest mean LD to the lowest mean LD in the flush. Note. CFU, colony-forming unit; LD, log density; C, connector; H, catheter hub; L, lumen; F, flush.

## Methods

### Connector classification and design features

The classification model categorizes most of the current globally marketed and tested devices based on their design components (Fig. 1).

### Study design

In total, 20 NC types were evaluated in this study (Fig. 2). Connector-catheter sets included a NC, hub, and catheter. Also, 33 independent experimental runs were performed. Each run included 4 replicates of the control NC, MicroClave (ICU Medical, San Clemente, CA), simultaneously with 2 different types of test NCs. Optimal experimental design dictates that a control NC be included in all runs; MicroClave was chosen as the control because it had the lowest bacterial transfer in the first few runs (Supplementary Fig. S1 online). Each of the 19 test NCs were studied in 3 runs with 4 replicates per run. Each run was completed over 5 days. On each day, there were 2 inoculations of ∼10^6^ CFU/mL *Staphylococcus aureus* (ATCC 6538) onto NC septum surfaces. An additional 1–2 replicates of each NC type were included to verify the quantity of bacteria that adhered to the septum after each inoculation. After the first inoculation, there were 6 flushes and 1 lock; after the second inoculation there were 9 flushes and 2 locks, for a daily total of 18 accesses. Moreover, 4 of these flushes were sampled, plated, and enumerated for bacteria. On each of the last 2 days, 2 connector-catheter sets for each NC type was destructively sampled, and the density of the bacterial biofilm attached to the intraluminal surfaces of the NC, hub, and catheter lumen were determined. This study, performed without disinfection protocols, ascertained the intrinsic risk of each NC. Additional experimental protocol details are included in the Supplementary Material (pages 4–6 and Supplementary Table S1 online).

### Study questions

The primary question of interest was whether there were differences among the 20 NCs in the number of bacteria transferred in the flush over 5 days. We also asked the following secondary questions: (1) Was there a difference in the number of biofilm bacteria in the NC, hub, and catheter lumen on days 4 and 5? (2) Was there an association between the number of biofilm bacteria in the NC, hub, and/or catheter lumen with the number of bacteria transferred in the flush? (3) Was there an association of 8 NC design factors with the number of biofilm bacteria in the NC or bacteria in the flush?

### Device design factors

Values for 8 design factors were determined and measured by ICU Medical engineers for 16 of the 20 NCs (Supplementary Tables S2 and S3 online). Some parameters could not be assessed because NCs were either unavailable on the global market (ie, SafeAccess, Planecta, and SafeTouch) or no longer sold in the United States (ie, Lily). These are the 8 design factors, their values, and the numbers of NCs with each value:

1. mechanism of access: luer-activated, 16; blunt cannula, 0
2. access portal: split septum, 11; surface septum, 5
3. flow path: mechanical valve, 8; internal cannula, 6; open path, 2
4. fluid displacement: neutral, 6; negative, 4; positive, 4; antireflux, 2
5. hydrodynamics: simple, 9; complex, 7
6. seal length including septum split length and/or circumference of surface septum: range, 0.066 cm to 1.575 cm; mean= 0.549 cm
7. flow path surface area: range, 0.981 cm^2^ to 15.419 cm^2^; mean = 5.503 cm^2^
8. flow path volume: range, 0.020 mL to 0.320 mL; mean = 0.136 mL.

### Statistical analyses

Bacterial transfer was measured as colony-forming units per flush (CFU/flush). The biofilm quantities in the NC, hub, and catheter segment were measured CFU/connector, CFU/hub, CFU/segment, respectively. The estimate of biofilm throughout the entire 55-cm catheter lumen from the processed 3-cm segment was calculated as CFU/lumen = 55/3 × CFU/segment. These densities were log-transformed to a log density (LD). For each of these responses, a daily mean LD was calculated for each replicate NC for every run. A linear mixed-effects model (LMM) was fit to the daily mean LDs across all 33 runs. Nested random effects included sample, run, and technician; fixed effects were day, connector, and their 2-way interaction. The inoculations of the surface control connectors were statistically equivalent at 95% confidence when NCs were tested side by side but were not statistically equivalent when not tested side by side (Supplementary Material, page 8 online). Therefore, a covariate for the daily mean bacterial LD of the surface inoculation was included. All results were reported as least-squares mean LDs. Additional details on LMMs for each of the flush, biofilm, and inoculation control data are reported in the Supplementary Material (pages 6–8 online). Tukey tests were used as follow-ups to all LMMs. Individual value, residual, and normal probability plots were used to check for outliers and to assess the normality and constant variance assumptions. Statistical tests and interaction plots were used to check for interactions. The LMMs fit to the flush and biofilm LDs were analyzed in R version 3.6.2 software (R Foundation for Statistical Computing, Vienna, Austria) and Minitab version 20 (Minitab, State College, PA). Conditional *R* ^2^ values were calculated using package *MuMIn*.^13^

## Results

### Bacteria in the flushes

**Fig. 4.**
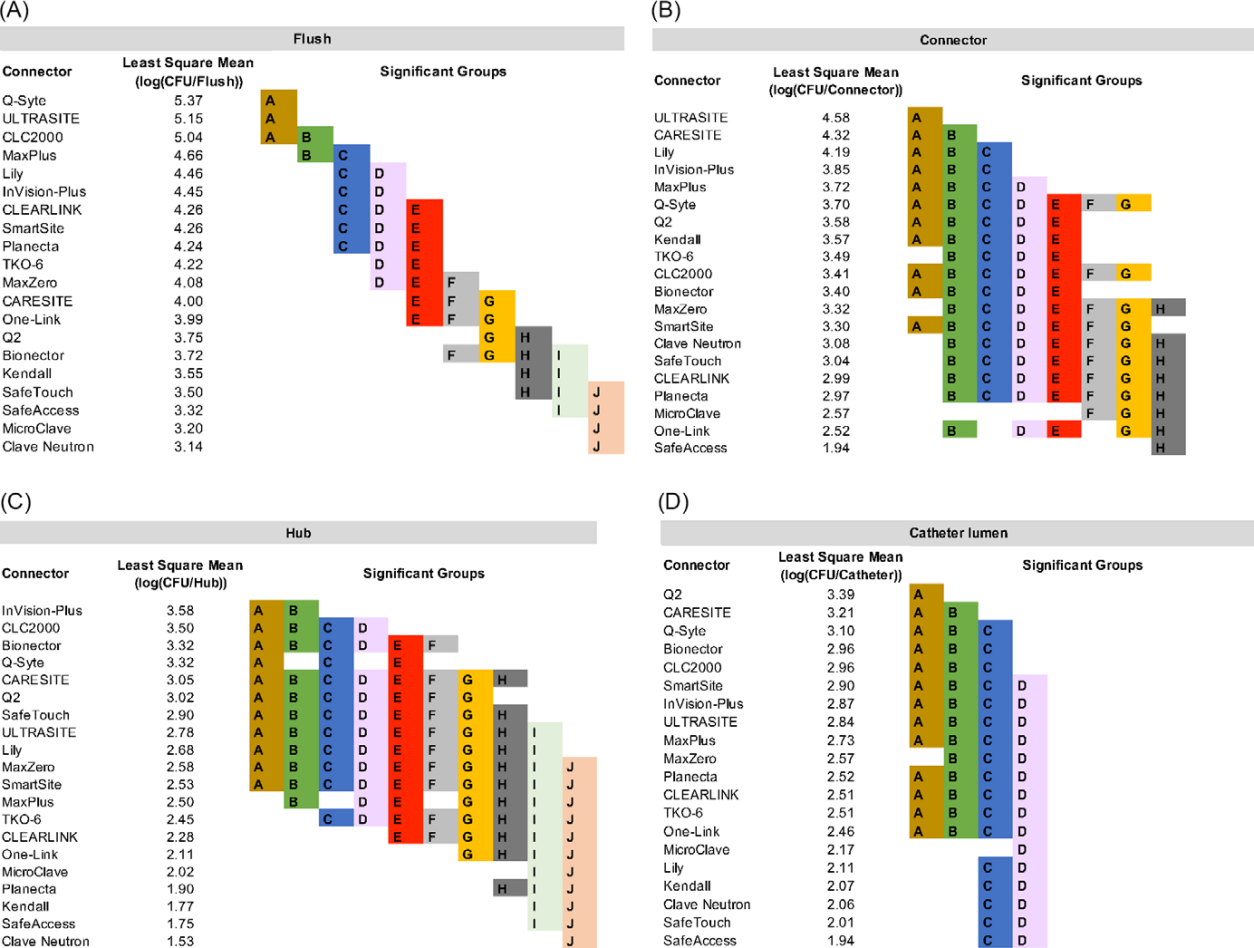
Least-squares mean bacterial log densities and significant groupings in (A) flushes, (B) needleless connectors, (C) catheter hubs, and (D) catheter lumen. NCs in different significant groups (indicated by A–J) are statistically significantly different (*P* < .05). Note. NC, needleless connector.

The mean bacterial LD in the flush for all NCs, pooled over 5 days, are presented in Figure 3B and compared in Figure 4A. Results for each run are shown in Supplementary Figure S1 (online). Clave Neutron (mean LD, 3.14) and MicroClave (mean LD, 3.20) had the lowest overall mean LD in the flush compared to any other NC (*P* < .05) except that there were no statistically significant differences between Clave Neutron, MicroClave, SafeTouch (mean LD, 3.32) and SafeAccess (mean LD, 3.50; *P ≥* .05). Q-Syte (mean LD, 5.37) and UltraSite (mean LD, 5.15) had the highest overall mean LD in the flush compared to all other NCs, except CLC2000 (mean LD, 5.04; *P <* .05).

**Fig. 5.**
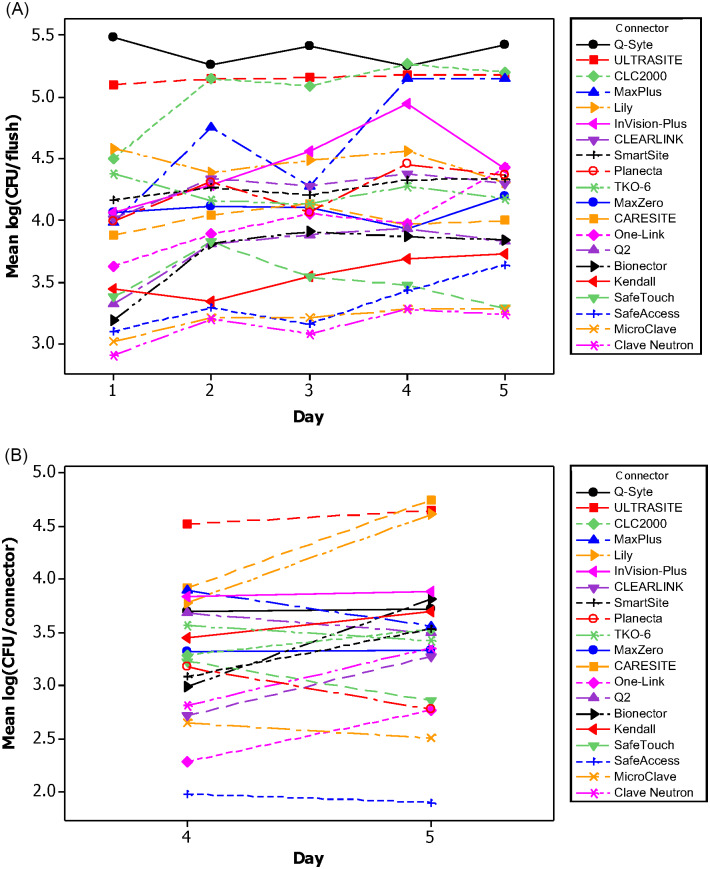
(A) The daily mean LD in the flush for each NC type over 5 days. (B) The daily mean LD in the connector on days 4 and 5. Note. CFU, colony forming unit; LD, log density; NC, needleless connector.

The trend over time of the mean LD in the flushes differed among NCs. Figure 5A shows the daily mean LD per flush among NCs averaged across all runs. Over 5 days, the daily mean LD of bacteria in the flush statistically significantly increased for Bionector (0.447/day), MaxPlus (0.243/day), InVision-Plus (0.175/day), and MicroClave (0.052/day; *P<* .05), indicating that both the number and daily increase of bacteria in the flushes were different for each NC.

### Biofilm bacteria in the NC, hub, and catheter lumen

**Fig. 6.**
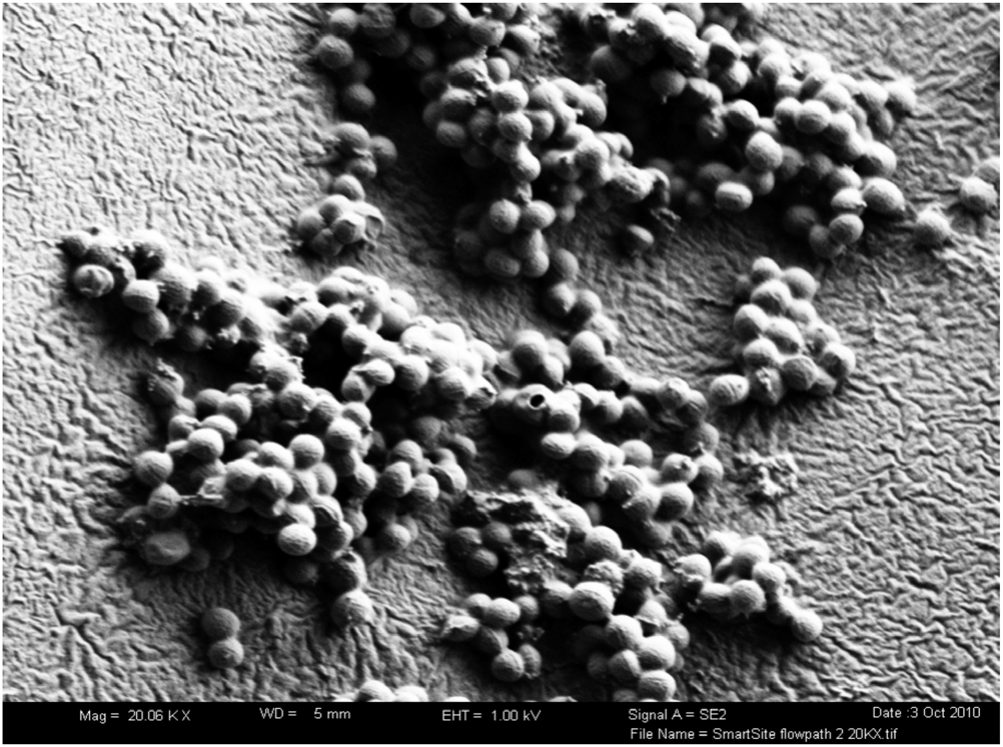
Scanning electronic microscope image of *Staphylococcus aureus* biofilm on the intraluminal surface in the flow path of the SmartSite needleless connector (magnification 20,000×).

Biofilm forms on the intraluminal surface of a NC (Fig. 6). The mean LDs of biofilm bacteria in the NC, hub, and catheter lumen for each NC type are presented in Figure 3B and compared in Figure 4B–D. Overall, the highest abundance of biofilm bacteria was in the NC (Fig. 3B) with the mean biofilm LD statistically significantly higher by 0.18 log on day 5 compared with day 4 (Fig. 5B). In the hub and catheter lumen, the mean LDs were not statistically significantly different on day 5 compared with day 4. Results for each experimental run are shown in Supplementary Figures S2–S4 (online).

### Association of biofilm in the NC, hub, and catheter lumen with flushes

**Fig. 7.**
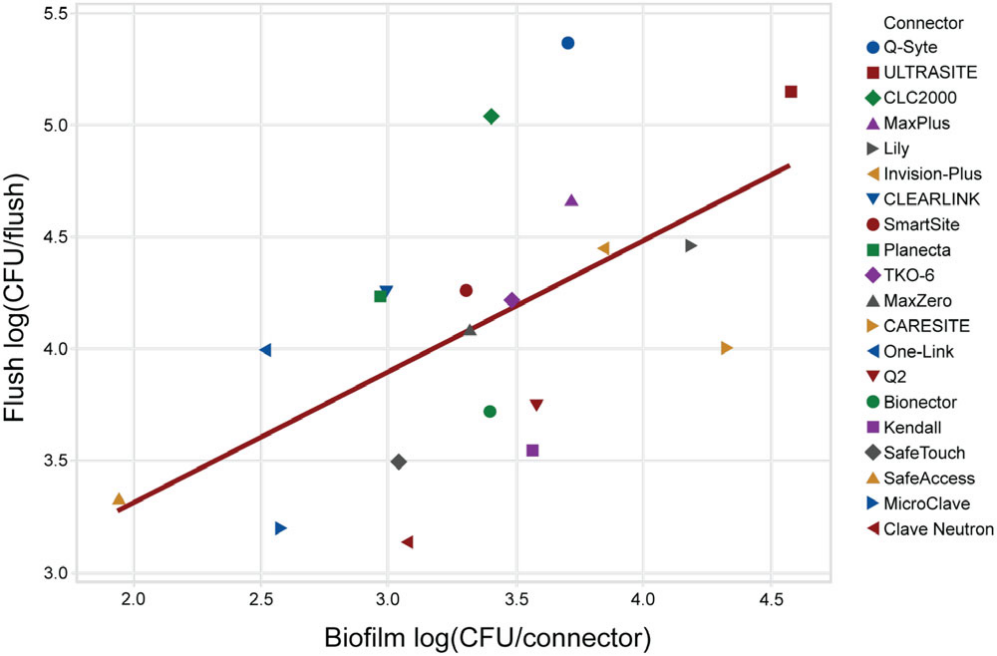
Association of the least-squares mean LDs of the biofilm bacteria in the connector and bacteria in the flush. Linear regression demonstrated an increase in bacteria in the flush with increasing biofilm bacteria in the connector. Note. LD, log density.

NC biofilm was the best predictor of bacteria in the flush among the 3 biofilm measures evaluated (NC, hub, and catheter lumen). After adjusting for surface inoculation, NC biofilm was the only statistically significant predictor of bacteria in the flush in both univariate (*P* = .037) and multivariable analyses (*P <* .0005) (Fig. 7). A principal component analysis further showed that NC biofilm had a different signature that strongly correlated with the flush, whereas the hub and catheter lumen were highly correlated. Regression equations are provided in the Supplementary Material (page 7 online).

### Association of design factors with biofilm in the NC and bacteria in the flush

The single most important NC design factor was flow path. When the flow path was through an internal cannula, there was ∼1 log (90%) reduction of bacteria in the flush (*R* ^2^ = 49%) and a 0.5–2 log (68%–99%) reduction in the biofilm bacteria in the NC (*R* ^2^ = 34%) on average compared to mechanical valve or open path designs. These substantial reductions in bacteria in the flush and NC biofilms remained associated with the flow path, even after accounting for all other design factors.

Displacement and hydrodynamics were independently associated with reductions of bacteria in the flush. On the average, NCs with neutral displacement had ∼1 log reduction (*R* ^2^ = 46%) compared to negative and positive displacements, and NCs with simple hydrodynamics had ∼1 log reduction (*R* ^2^ = 45%) compared to complex hydrodynamics. However, when the effects of all design factors were considered simultaneously, displacement and hydrodynamics were not associated with bacteria in the flush. This finding suggests the lower bacterial transfer initially seen with neutral displacement and simple hydrodynamic NCs may be attributable to other design features such as flow path, seal length, surface area, and volume.

The 5 categorical design factors together (ie, flow path, displacement, hydrodynamics, access, and antireflux) and the 3 continuous design factors (ie, seal length, flow path surface area, and flow path volume) best explained bacteria in the flush (*R* ^2^ = 65%) and biofilm in the connector (*R* ^2^ = 48%).

## Discussion

Current practice guidelines indicate that the optimal NC design for CRBSI prevention is unknown.^10,11^ Although several design features have been associated with BSIs, none have been studied for their relative contribution to the risk.^14^ Recent investigation of the clinical impact of NC design maintains that analyses of microbial ingress, antiseptic methods, and biofilm formation in different designs are needed to address CRBSIs.^1^ To our knowledge, this is the most comprehensive study of NC design, intraluminal biofilm formation, and bacterial transfer risk to date. Our study was performed over 5 years with 33 independent experimental runs using 20 nondisinfected NCs to determine differences in bacterial transfer in the flush and intraluminal biofilm colonization in the connector-catheter system, allowing an assessment of contributing design factors.

Since the clinical introduction of NCs, CRBSIs have been linked to their use. Intraluminal biofilms were first detected in NCs harvested from patients and tested at the Centers for Disease Control and Prevention (CDC) in 2001.^15^ Outbreaks have been reported with each design generation^1^; moving from the original split septum/blunt cannula to mechanical valves differentiated by negative, positive, and neutral displacement.^14^ Early investigation implicated negative and positive displacement mechanical valve NCs to be associated with increased BSIs.^16^ Research has since suggested that differences in contamination risk among NCs (even with disinfection) are not specific to the displacement categorization as either positive, negative, or neutral.^17^ A recent literature review also reported that BSI rates were variable for different displacement categorizations.^1^

This clinically simulated in vitro study was designed to represent a common treatment schedule for intermittent intravenous infusion, and was modified from standardized, controlled protocols used to investigate NC biofilm development.^15,18^ Previous investigators mostly used in vitro models to compare bacterial transfer in the flush with variable protocols, some including NC disinfection.^17,19,20^

NC septum disinfection prior to each access is a recognized critical step to prevent microbial transfer.^21^ However, the most effective disinfection method remains unknown as no study to date has tested against septum surface biofilms, even though significant biofilm accumulation has previously been observed on NC septums and internal surfaces using scanning electron microscopy.^22^ Compliance with clinical NC disinfection is variable (12%–100%),^23,24^ with poor adherence to disinfection protocols and techniques.^24^ Accordingly, microbial burden on NC septum surfaces prior to disinfection has ranged from 10^1^ to 10^5^ CFU in up to 67% of NCs.^25–27^ This is likely an underestimate because simplistic culturing techniques used in these studies typically yield low retrieval rates.^18,28^ Thus, more vigorous methods of sonication and vortex, as used in this study, are necessary to remove and disaggregate surface-attached biofilm cells.^17,29^ Hence, this study was performed without disinfection, and with a high inoculum (10^6^ CFU), to simulate both the microbial burden found clinically and the worst-case scenario of NC disinfection noncompliance. Omission of the disinfection step was also intended to robustly ascertain performance differences among NCs and to compare the intrinsic risk of each NC in the absence of proper disinfection.

Our results demonstrated several key findings, among dispelling displacement categorization as the sole predictor of CRBSIs. First, flow path was the most important design factor; the internal cannula was associated with less bacterial transfer and biofilm formation. Second, neutral displacement and simple hydrodynamics were associated with reduced NC biofilm and bacterial transfer in the flush. Complex designs with internal irregularities can disrupt laminar flow, which can cause high velocity and turbulence, contributing to biofilm formation.^30–34^ Third, variation in access portal seal length, surface area, and volume influence differences seen among NCs with similar and dissimilar displacement and hydrodynamic characteristics. This likely explains why all 8 factors simultaneously best predict bacterial transfer and biofilm formation and that displacement classification alone can be misleading. Lastly, biofilm bacteria were most abundant in the NC compared to the hub and lumen, which was also the only significant predictor of bacteria in the flush.

The CDC recommends NC exchange at least every 96 hours or according to the manufacturer’s instructions.^35^ Our analyses support this, given the high levels of biofilm on the NC, hub, and catheter lumen by 72 hours. However, our study also demonstrates that some NCs require more frequent exchanges, given that some NCs allow more bacteria through the connecter over time compared to others. Even after 5 days, bacterial counts in the flush among some NCs never reached the high levels that were observed after 24 hours in other NCs (Fig. 5A). The substantial differences in the daily bacterial transfer rate and the increase in bacteria over time for some NC types warrant reconsideration of NC exchange recommendations. However, it is important to note that NC replacement does not affect biofilm in the hub or catheter, as these distal components will continue to harbor biofilm.

Current IDSA Clinical Practice Guidelines recommend culturing long-term catheter insertion site and hub to rule out the catheter as the infection source for CRBSIs,^36^ but this is rarely practiced due to the complexity and risk of obtaining hub cultures. In a series of studies, researchers investigated the efficacy of insertion-site skin cultures and NC flush sonication cultures in the diagnosis of CRBSI to show that the combination of skin culture and NC quantitative cultures (>1,000 CFU/connector) can be used to rule out catheter colonization and CRBSI.^37^ Our results support these findings because NC biofilm was the best predictor of bacterial transfer in the flush. Moreover, in our study, MicroClave, Clave Neutron, SafeTouch and SafeAccess NCs generated substantially lower rates of bacterial transfer. This finding may explain a reduced relative risk of CLABSI in hospitals using Clave technologies compared to hospitals using other NCs.^38^

This study had several limitations. First, the study design may not have represented all possible uses of NCs because clinical access frequencies and patterns vary greatly. Second, blinding study personnel to NCs was not possible due to visual distinguishability, which potentially introduces bias, and procedures were standardized to minimize this bias. Third, the use of lower inoculum and/or other organisms may produce dissimilar results. Fourth, although we focused primarily on design factors most likely to influence biofilm formation based on prior research, other factors may exist. Material type would be one to consider; however, it would be difficult to assess because septum, housing, and internal components use different materials in different NCs. Lastly, additional clinical studies are warranted to assess real-world risk for CRBSI with different NC types.

Overall, our findings have demonstrated that NC choice can be an important CRBSI risk reduction strategy. Bacterial transfer and biofilm formation through the connector-catheter systems were statistically significantly different among NCs with different designs. Furthermore, biofilm in the NC was predictive of bacterial transfer in the flush. The NC designs associated with the least bacterial transfer and biofilm formation were NCs with a split septum, minimal seal length, internal cannula, low surface area and volume, neutral displacement, and simple hydrodynamics of the flow path. Although NC disinfection remains critically important, this research highlights the need to choose NCs with the least risk of biofilm formation and bacterial transfer to reduce CRBSIs.

## Supplementary material

For supplementary material accompanying this paper visit https://doi.org/10.1017/ice.2023.60. click here to view supplementary material
