# Supplementary material for: Bacterial transfer and biofilm formation in needleless connectors in a clinically simulated in vitro catheter model
Source: Infect Control Hosp Epidemiol. 2023 Apr 24;44(11):1760–8. doi: 10.1017/ice.2023.60 (PMC10665874; doi:10.1017/ice.2023.60)
Supplement: Supplementary file 1 [file S0899823X23000600sup001.docx]

**Supplemental Materials**

**Bacterial transfer and biofilm formation in needleless connectors in a clinically simulated in vitro catheter model**

**Authors:** Marcia Ryder, PhD MS RN^1^, Elinor deLancey-Pulcini^2^, PhD, Albert E Parker^2,3^, PhD, Garth James, PhD^2^

**Affiliations:**

^1^ Ryder Science, Brentwood, TN

^2^ Center for Biofilm Engineering, Montana State University, Bozeman, MT

^3^ Department of Mathematical Sciences, Montana State University, Bozeman, MT

##

**Table of Contents**

[Study Protocol 4](#_Toc120101783)

[Inoculation 4](#_Toc120101784)

[Connector testing 4](#_Toc120101785)

[Destructive sampling 5](#_Toc120101786)

[Statistical Analyses 6](#_Toc120101787)

[Bacteria in the flushes 6](#_Toc120101788)

[Biofilm bacteria in the NC, hub, and catheter lumen 6](#_Toc120101789)

[Association of biofilm in the NC, hub, and catheter lumen with flushes 7](#_Toc120101790)

[Effect of design factors 8](#_Toc120101791)

[Equivalence of connector septum inoculum 8](#_Toc120101792)

[Supplemental Tables 9](#_Toc120101793)

[Table S1. Descriptive Protocol Table 9](#_Toc120101794)

[Table S2. Summary table of each NC’s seal length, flow path surface area and flow path volume 12](#_Toc120101795)

[Table S3. Detailed seal length measurements and connector access portal image for each NC 14](#_Toc120101796)

[Supplemental Figures 23](#_Toc120101797)

[Figure S1. Mean LDs per flush for each connector plotted across runs 23](#_Toc120101798)

[Figure S2. Run mean biofilm LD attached to the connector plotted across runs 24](#_Toc120101799)

[Figure S3. Run mean biofilm LD attached to the hub plotted across runs 25](#_Toc120101800)

[Figure S4. Run mean biofilm LD attached to the segment plotted across runs 26](#_Toc120101801)

[Figure S5. Run mean surface inoculation LDs (averaged across the two inoculations on each day and all days) plotted across runs 27](#_Toc120101803)

# **Study Protocol**

## **Inoculation**

Experiments were conducted using *Staphylococcus aureus* (ATCC 6538), a clinical strain commonly used for biofilm testing. Overnight cultures were grown from frozen stocks in full-strength Tryptic Soy Broth (TSB) at 37°C. A fresh inoculum was prepared at the start of each day, and inoculum density was confirmed by serial dilution and plating onto Tryptic Soy Agar (TSA) plates. Daily procedures (**Table S1**) began with (a) the inoculation of each study and surface control connector (four test and two surface control connectors of each type) by dipping a sterile cotton-tipped swab into the inoculum and “painting” the surface of the septum applying approximately 10^6^ colony forming units (CFU/inoculation) of *S.* *aureus*.

## **Connector testing**

On day one, the test units consisting of a test connector attached to a 5 Fr, 55 cm peripherally inserted central catheter (PICC) (PFM Medical, Inc., Carlsbad, CA) were assembled. Each septum of each test connector and one to two additional surface control connectors were inoculated (b). The (c) inoculated connectors were air-dried for 30 minutes; (d) the catheter-connecter sets were flushed with 3.0 ml of sterile 0.9% normal saline (NS); (e) the flushes were collected and plated; (f) catheter-connector sets were flushed two more times with 3.0 ml NS; (g) catheter-connector sets were locked with 2.0 ml sterile Brain Heart Infusion Broth (BHI) in lieu of heparin for one hour; (h) the catheter-connector sets were flushed three more times with 3.0 ml NS; (i) the last 3.0 ml flush was collected and plated (CFU/flush). Steps (d)-(g) and (h) simulated the Saline-Administration-Saline-Heparin (SASH) flushing/locking method recommended for intermittent medication infusion within open-ended catheters.^1^

Steps (b) through (i) were repeated for each NC, only the final flush, after nine flushes and two locks, was plated. The agar plates were incubated overnight at 37°C, the colony forming units (CFU) were counted and expressed as CFU/flush. The entire inoculation, lock, and flush procedure was repeated over five days. On day 4 (representing 96 hours) and day 5, two of the catheter-connector sets for each connector type were removed from the test and destructively sampled.

## **Destructive sampling**

The connectors were sonicated and flushed with 1.0 ml sterile phosphate-buffered saline (PBS) into a sterile conical vial. The flush solutions were serially diluted and plated onto Tryptic Soy Agar (TSA) plates. The catheters were flushed with 2.0 ml NS into a sterile conical vial, serially diluted, and plated onto TSA. The hubs were aseptically cut from the catheter, surface-disinfected with 95% ethanol, and placed in a vial of 10 ml sterile PBS. The tubes were vortexed for ten seconds, sonicated in a bath sonicator (csu-3, Tuttnauer) for two minutes, and vortexed another ten seconds to remove and disaggregate bacterial cells. The cell suspensions were serially diluted and plated.

The 3.0 cm catheter segment was aseptically cut 2.0 cm distal to the hub, surface-disinfected with 95% ethanol, and placed in a separate vial of 10.0 ml sterile PBS. The attached cells were scraped from the intraluminal surface of the catheter segments using a sterile wire. The wire and catheter lumen were rinsed with 1.0 ml of PBS. This process was repeated for a total of three times. The tubes were then vortexed, sonicated, vortexed and the cell suspension serially diluted and plated.

All plates were incubated overnight at 37°C. The CFUs were counted and reported as CFU/connector or CFU/hub or CFU/segment. The estimate of biofilm throughout the entire 55cm catheter lumen from the processed 3cm segment was calculated as CFU/lumen = 55/3 × CFU/segment.

# **Statistical Analyses**

## **Bacteria in the flushes**

A daily mean LD was calculated for each of the three to five replicates of each connector type for every run. To analyze the flushes, a linear mixed-effects model (LMM) was fit to the daily mean flush LDs across all 33 runs simultaneously. There were three nested random effects: sample, run and technician. The fixed effects were day, connector, and their two-way interaction. A covariate for the daily mean bacterial LD of the surface inoculation was also included to adjust for slight differences in inoculation levels across the runs.

## **Biofilm bacteria in the NC, hub, and catheter lumen**

To compare bacterial attachment among the connector, hub, and catheter segment, an LMM was fit separately to each response across all 33 runs simultaneously with nested random effects for run and technician, fixed effects for day, connector and the two-way interaction, and a covariate for the overall run-mean surface inoculation level for each connector type. Across all 33 runs and 20 connector types, a predictive model assessed whether some combination of the daily mean LD in the hub, connector or catheter lumen measured on days 4 and 5 could predict the mean LD of bacteria in the flush on days 4 and 5. This was accomplished by modeling the daily mean flush LD with an LMM with connector model as a fixed effect. The nested random effects were run and technician while the run-mean surface inoculation control, day, and the interaction between day and connector type served as covariates. Bacterial LDs in the NC, hub, and catheter lumen were the predictors in this model and were added one-by-one in separate analyses as covariates, each time including the two-way interaction with connector. A fourth model included all predictors simultaneously as covariates but did not contain any two-way interactions between predictors. Finally, a principal component analysis was performed on the correlation matrix of the daily mean LD in the connector, hub, catheter lumen, flush, and surface inoculation controls.

## **Association of biofilm in the NC, hub, and catheter lumen with flushes**

Biofilm in the NC was the best predictor of bacteria in the flush among the three biofilm measures evaluated: NC, hub and catheter lumen. This conclusion is supported by the following three analyses:

1. When assessing individually, after adjusting for surface inoculation controls, the biofilm in the NC was the only statistically significant predictor of bacteria in the flush (*P* = 0.037), yielding the regression equation: $\log[CFU/flush]=5.54+0.18 \log[CFU/connector]-0.293\log[CFU/innoculation]$
2. Biofilm in the NC was the only statistically significant predictor when each component was analyzed simultaneously as predictors of the flush (*P* < 0.0005), yielding the regression equation: $log[CFU/flush]=9.01+0.18 \log[CFU/connector]-0.04 \log[CFU/hub]-0.08 \log[CFU/lumen]-0.38 \log[CFU/inoculation]$
3. A principal component analysis showed that the catheter lumen and hub were highly correlated, whereas the NC had a markedly different signature that strongly correlated with the flush.

## **Effect of design factors**

The effect of the eight design factors was investigated with an LMM with the random effects as described above. The “best” parsimonious model was determined by conditional Akaike information criterion (AIC), which was calculated using package cAIC4^2^, and bias-corrected Bayesian information criterion (BIC) and conditional *R*^2^ were calculated using package MuMIn.^3^ All models included a covariate for day and a covariate for the daily mean inoculation level. Design factors were added in a stepwise manner, and then all eight simultaneously in an additive model, with and without all 2-way and 3-way interactions among surface area, volume and seal length.

## **Equivalence of connector septum inoculum**

An LMM was fit to the inoculation surface LDs of the surface control connectors with fixed effects for inoculations nested in day (two per day), connector and the two interactions: run, and technician served as random effects. Tukey 90% confidence intervals were constructed to make claims of statistical equivalence at 95% confidence. The run average log (CFU/connector) of the inoculum density across the 20 NC types and 33 runs ranged from 5.71 (MicroClave in run 22) to 7.32 (InVision-Plus in run 18) (**Figure S5**). Since the mean differences in log (CFU/connector) as large as 0.5 are considered negligible and not of practical importance^4-7^, then each set of three runs with the same set of NCs yielded surface inoculations were statistically equivalent. In other words, the same bio-challenge was presented to all NCs that were tested side-by-side in the same runs. However, the data did not establish statistical equivalence for NCs that were not run side-by-side due to run mean differences in inoculation up to 0.78 log (CFU/connector). Therefore, the comparisons were adjusted for the LD of surface inoculation controls when comparing NC types.

# **Supplemental Tables**

## **Table S1.** Descriptive Protocol Table

| **Day 1** | **Day 2 and Day 3** | **Day 4 and Day 5** |
| --- | --- | --- |
| Prepare 4 MicroClave, 4 Type A and 4 Type B catheter-connector sets |  |  |
| Inoculate 10^6^ CFU/connector plus 1-2 extra connectors each | Inoculate 10^6^ CFU/connector plus 1-2 extra connectors each | Inoculate 10^6^ CFU/connector plus 1-2 extra connectors each |
| Dry 30 minutes | Dry 30 minutes | Dry 30 minutes |
| Plate each connector surface controls | Plate each connector surface controls | Plate each connector surface controls |
| Flush 3.0 ml saline | Flush 3.0 ml saline | Flush 3.0 ml saline |
| Plate flush | Plate flush | Plate flush |
| Flush 3.0 ml saline | Flush 3.0 ml saline | Flush 3.0 ml saline |
| Flush 3.0 ml saline | Flush 3.0 ml saline | Flush 3.0 ml saline |
| **Lock 1% BHI 1 hr** | **Lock 1% BHI 1 hr** | **Lock 1% BHI 1 hr** |
| Flush 3.0 ml saline | Flush 3.0 ml saline | Flush 3.0 ml saline |
| Flush 3.0 ml saline | Flush 3.0 ml saline | Flush 3.0 ml saline |
| Flush 3.0 ml saline | Flush 3.0 ml saline | Flush 3.0 ml saline |
| Plate the last flush | Plate the last flush | Plate the last flush |
| Inoculate 10^6^ CFU/connector on the sets plus 1-2 extra connectors each | Inoculate 10^6^ CFU/connector on the sets plus 1-2 extra connectors each | Inoculate 10^6^ CFU/connector on the sets plus 1-2 extra connectors each |
| Dry 30 minutes | Dry 30 minutes | Dry 30 minutes |
| Plate each connector surface controls | Plate each connector surface controls | Plate each connector surface controls |
| Flush 3.0 ml saline | Flush 3.0 ml saline | Flush 3.0 ml saline |
| Plate flush | Plate flush | Plate flush |
| Flush 3.0 ml saline | Flush 3.0 ml saline | Flush 3.0 ml saline |
| Flush 3.0 ml saline | Flush 3.0 ml saline | Flush 3.0 ml saline |
| **Lock 1% BHI 1 hr** | **Lock 1% BHI 1 hr** | **Lock 1% BHI 1 hr** |
| Flush 3.0 ml saline | Flush 3.0 ml saline | Flush 3.0 ml saline |
| Flush 3.0 ml saline | Flush 3.0 ml saline | Flush 3.0 ml saline |
| Flush 3.0 ml saline | Flush 3.0 ml saline | Flush 3.0 ml saline |
| **Lock 1% BHI 1 hr** | **Lock 1% BHI 1 hr** | **Lock 1% BHI 1 hr** |
| Flush 3.0 ml saline | Flush 3.0 ml saline | Flush 3.0 ml saline |
| Flush 3.0 ml saline | Flush 3.0 ml saline | Flush 3.0 ml saline |
| Flush 3.0 ml saline | Flush 3.0 ml saline | Flush 3.0 ml saline |
| Plate the last flush | Plate the last flush | Plate the last flush |
|  |  | Sample 2 MicroClave , 2 Type A and 2 Type B catheter-connector sets |

## **Table S2**. Summary table of each NC’s seal length, flow path surface area and flow path volume

| **#** | **Connector Name** | **Access Portal** | **Mean Seal Length (cm)** | **Flow Path Surface Area (cm^2^)** | **Unactivated Flow Path Volume (mL)** |
| --- | --- | --- | --- | --- | --- |
| 1 | Clave Neutron (NC100) | Split Septum | 0.090 | 4.452 | 0.10 |
| 2 | MicroClave (MC100) | Split Septum | 0.066 | 1.426 | 0.04 |
| 3 | SafeAccess | Split Septum | Not Available | 2.658 | 0.08 |
| 4 | SafeTouch | Split Septum | Not Available | 3.355 | 0.06 |
| 5 | Kendall | Split Septum | 0.156 | 4.277 | 0.12 |
| 6 | Bionector | Split Septum | 0.201 | 1.155 | 0.02 |
| 7 | Q2 | Split Septum | 0.249 | 1.516 | 0.10 |
| 8 | One-Link | Split Septum | 0.135 | 5.606 | 0.08 |
| 9 | CARESITE | Surface Septum | 1.583 | 12.619 | 0.22 |
| 10 | MaxZero | Surface Septum | 1.340 | 9.529 | 0.19 |
| 11 | TKO-6 | Split Septum | Not Available | 5.387 | 0.15 |
| 12 | Planecta | Split Septum | Not Available | 2.065 | 0.17 |
| 13 | SmartSite | Split Septum | 0.257 | 2.271 | 0.11 |
| 14 | CLEARLINK | Split Septum | 0.161 | 3.019 | 0.25 |
| 15 | InVision-Plus | Split Septum | 0.069 | 0.981 | 0.03 |
| 16 | Lily | Surface Septum | Not Available | Not Available | Not Available |
| 17 | MaxPlus | Surface Septum | 1.357 | 15.387 | 0.32 |
| 18 | CLC2000 | Surface Septum | 1.280 | 3.258 | 0.06 |
| 19 | ULTRASITE | Surface Septum | 1.324 | 14.619 | 0.30 |
| 20 | Q-Syte | Split Septum | 0.358 | 2.587 | 0.08 |

Note: Properties of flow path, access portal, and anti-reflux valve were apparent upon examination of each connector, while fluid displacement was reported by the manufacturer. Hydrodynamics were informed through prior work and visual analysis of the fluid path behavior for connectors.^8^ The seal length was measured using a Smartscope MVP (Optical Gaging Products, Rochester, NY) and fitting either a circle or line to the seal for each connector (n=3). Seal length (perimeter) was then calculated from the measured diameter, or the length reported for slit type connectors. Values represent the mean seal length. The flow path surface area and volume were calculated by recreating a 3D model of each connector and using the software (SolidWorks) to calculate the surface area or volume in contact with the fluid.

*Seal length was measured using TKO-6P. All other data (flow path surface area and flow volume) were collected for TKO-6. Of note, the slit on both connectors is the same, and the analysis was unchanged.

## **Table S3**. Detailed seal length measurements and connector access portal image for each NC

| **#** | **Manufacturer** | **Connector Name** | **Connector Label** | **Access Portal** | **Seal Length**  **(cm)** | **Connector Access Portal Image** |
| --- | --- | --- | --- | --- | --- | --- |
| 1 | ICU Medical | Clave Neutron | 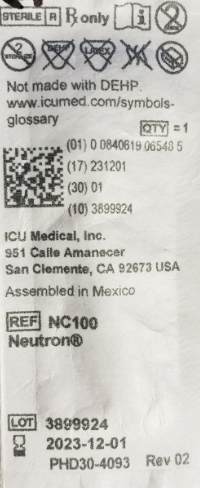 | Split Septum | 0.0853  0.0836  0.1008 | 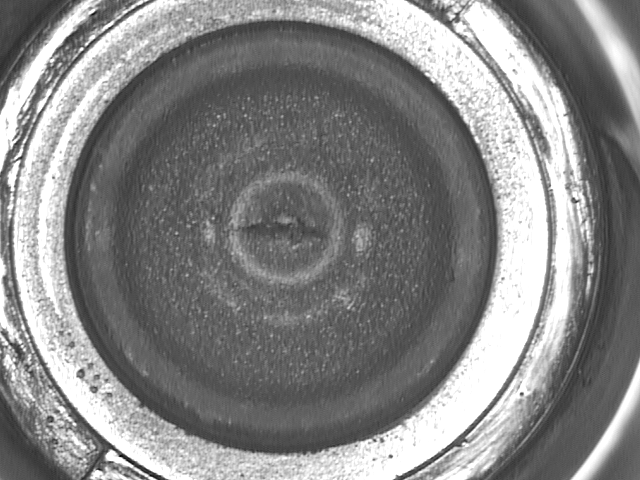 |
| 2 | ICU Medical | MicroClave | 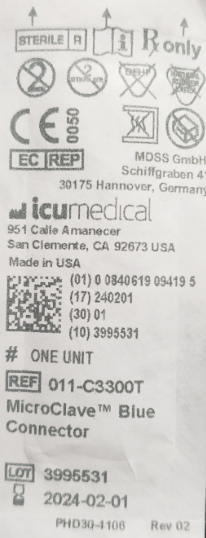 | Split Septum | 0.0714  0.0640  0.0635 | 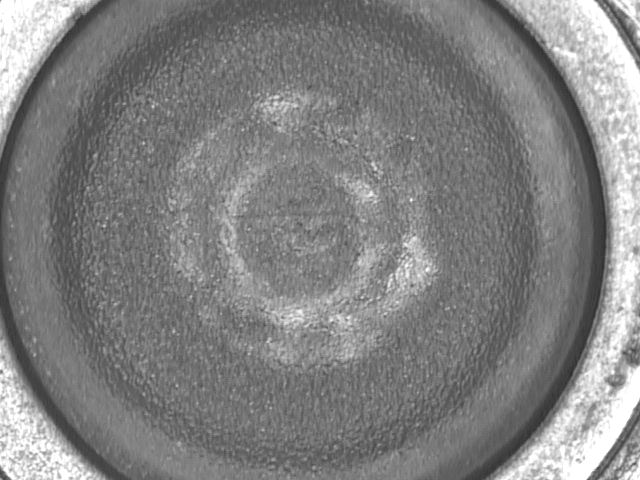 |
| 3 |  | SafeAccess |  |  |  |  |
| 4 |  | SafeTouch |  |  |  |  |
| 5 | Covidien | Kendall | 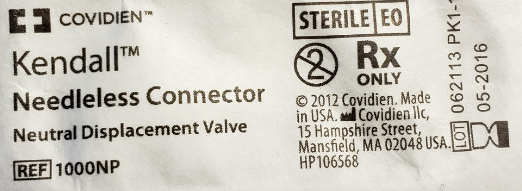 | Split Septum | 0.1222  0.1344  0.2103 | 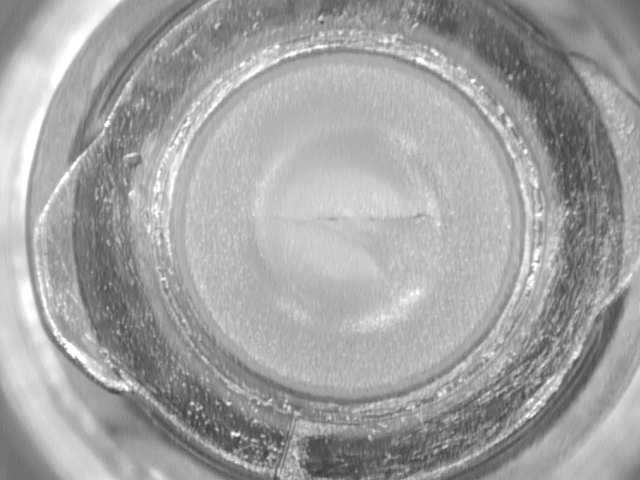 |
| 6 | Vygon | Bionector | 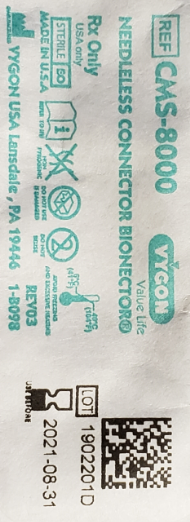 | Split Septum | 0.1666  0.2319  0.2047 | 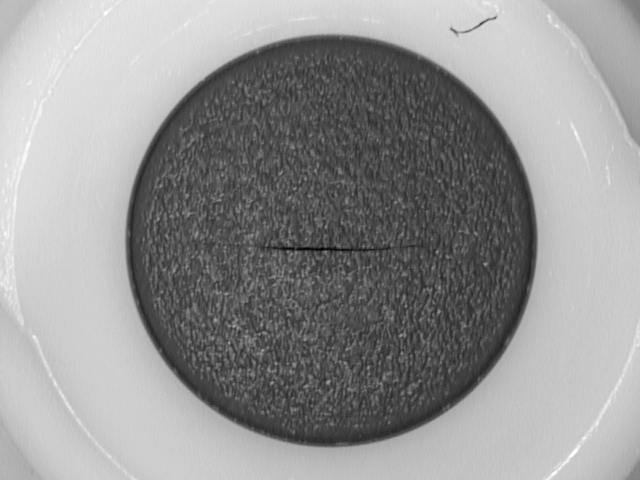 |
| 7 | Quest Medical | Q2 | 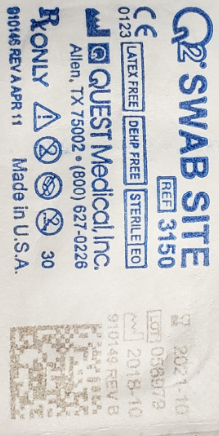 | Split Septum | 0.2814  0.2220  0.2446 | 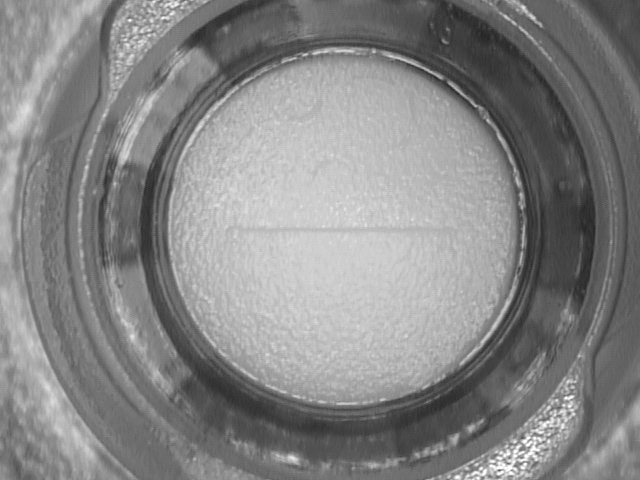 |
| 8 | Baxter | ONE-LINK | 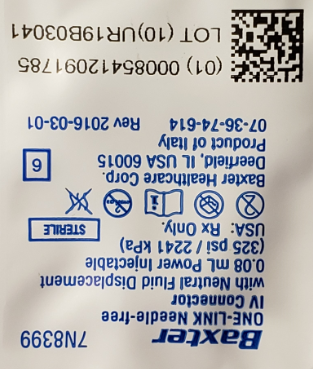 | Split Septum | 0.1412  0.1351  0.1273 | 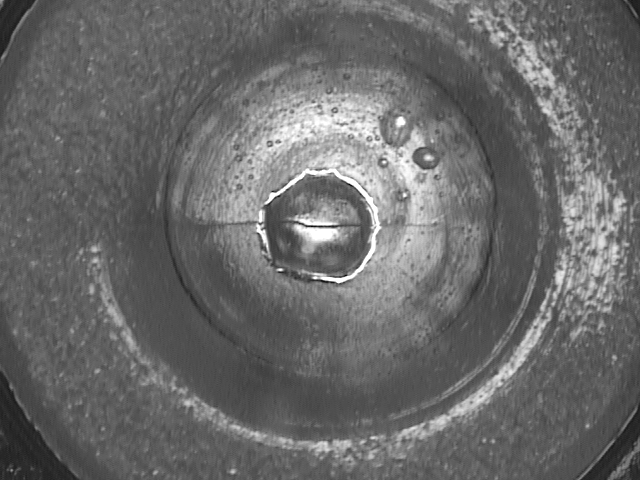 |
| 9 | B. Braun | CARESITE | 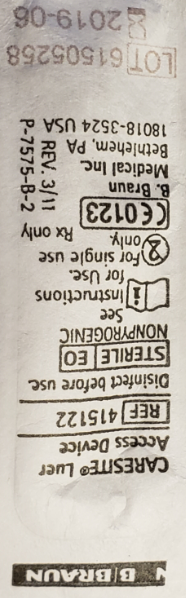 | Split Septum  +  Surface Septum | 0.2418  0.2507  0.2497  Dia. 0.4252  Dia. 0.4265  Dia. 0.4239 | 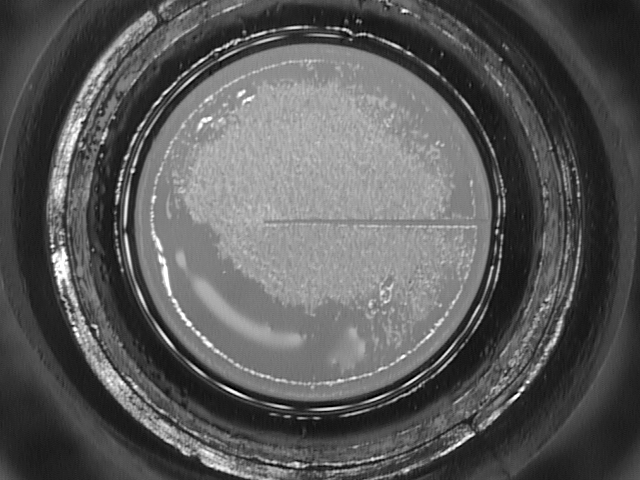 |
| 10 | BD | MaxZero | 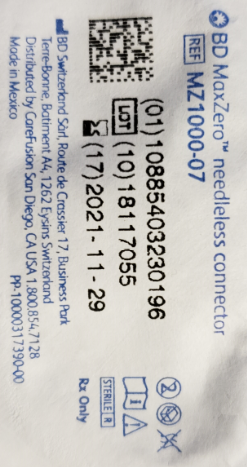 | Surface Septum | Dia. 0.4257  Dia. 0.4270  Dia. 0.4272 | 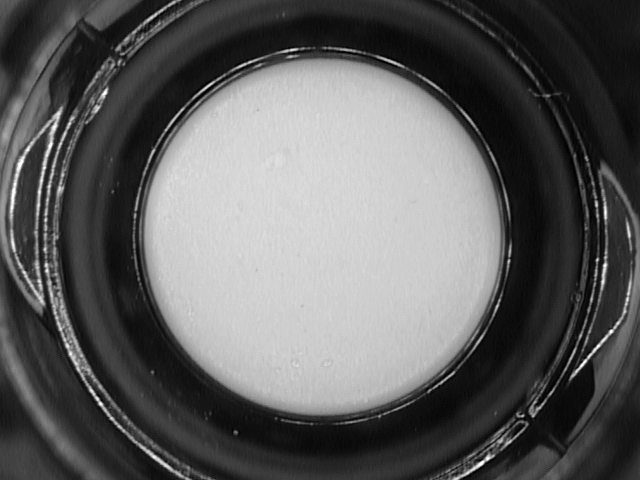 |
| 11 | Nexus Medical | TKO-6P | 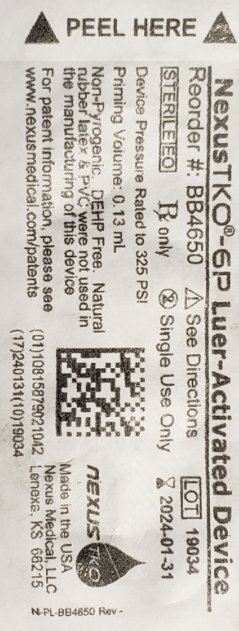 | Split Septum | 0.1168*  0.1463*  0.1547* | 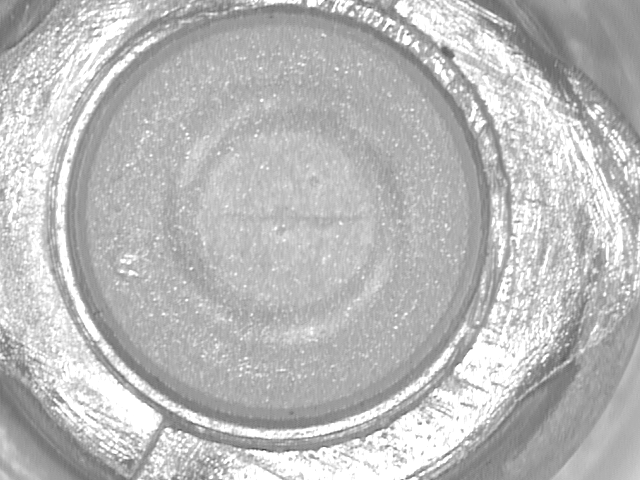 |
| 12 |  | Planecta |  |  |  |  |
| 13 | BD | SmartSite | 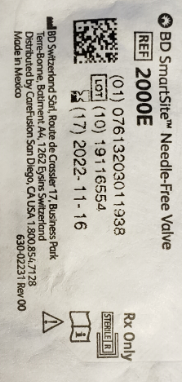 | Split Septum | 0.2700  0.2553  0.2466 | 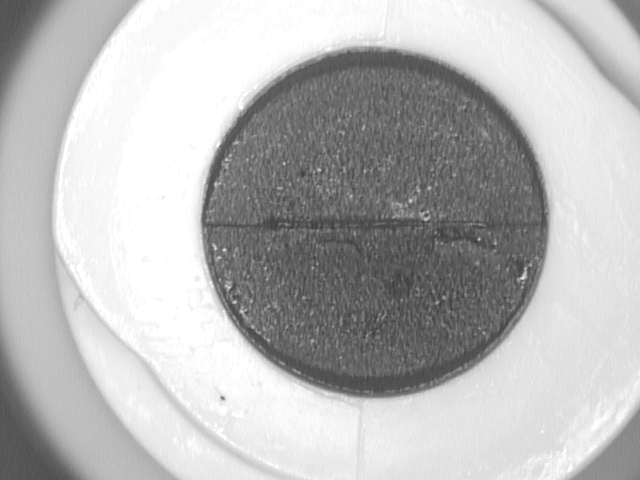 |
| 14 | Baxter | CLEARLINK | 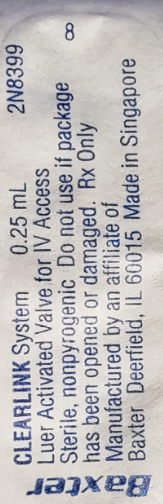 | Split Septum | 0.1712  0.1582  0.1537 | 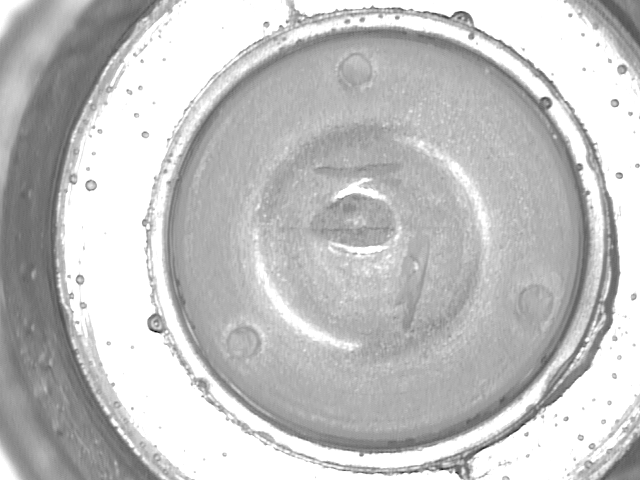 |
| 15 | RyMed | InVision-Plus | 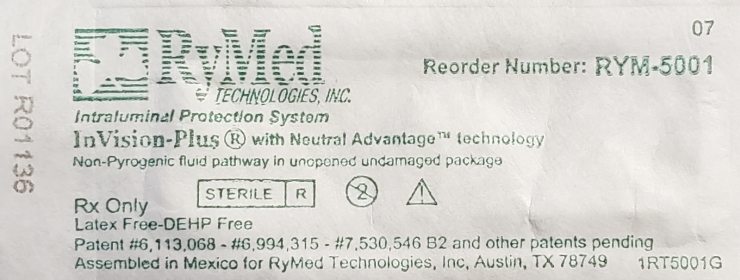 | Split Septum | Connectors on hand were expired and seal length could not be measured | 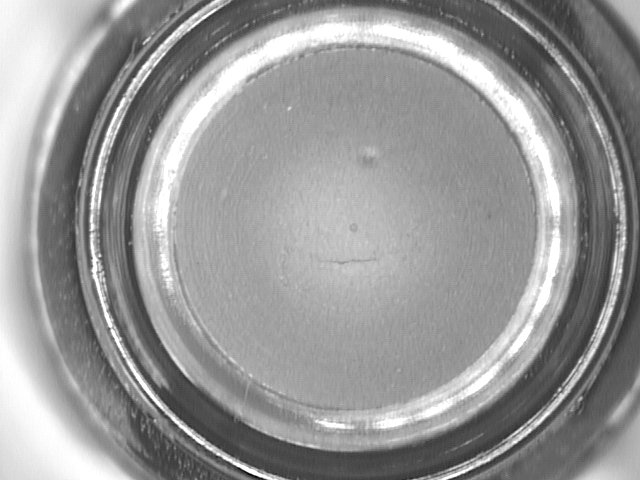 |
| 16 |  | Lily |  |  |  |  |
| 17 | BD | MaxPlus | 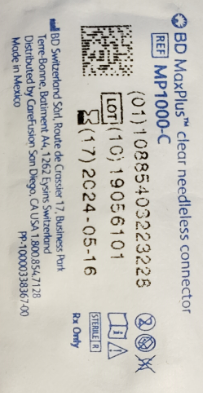 | Surface Septum | Dia. 0.4323  Dia. 0.4313  Dia. 0.4323 | 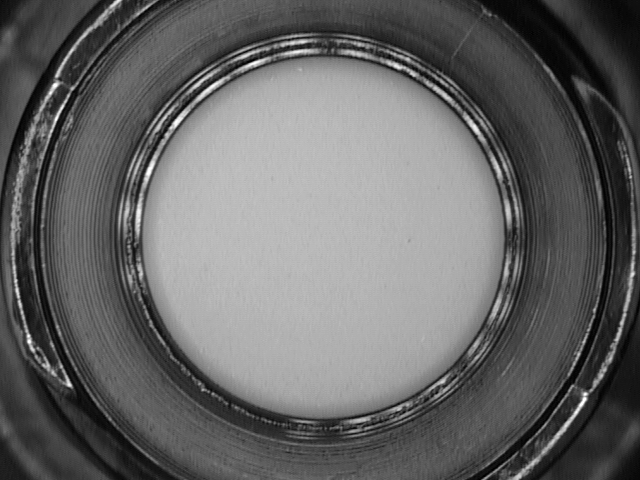 |
| 18 | ICU Medical | CLC2000 | Not Available | Surface Septum | Dia. 0.4079  Dia. 0.4059  Dia. 0.4084 | 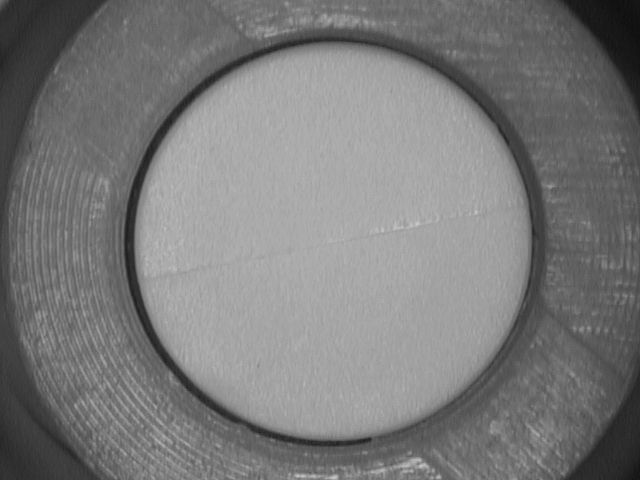 |
| 19 | B.Braun | ULTRASITE | 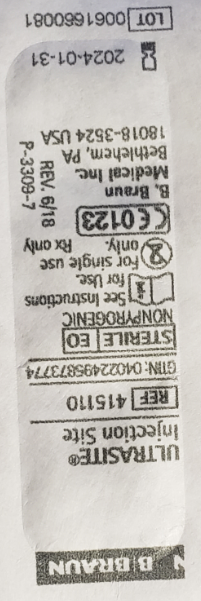 | Surface Septum | Dia. 0.4221  Dia. 0.4206  Dia. 0.4216 | 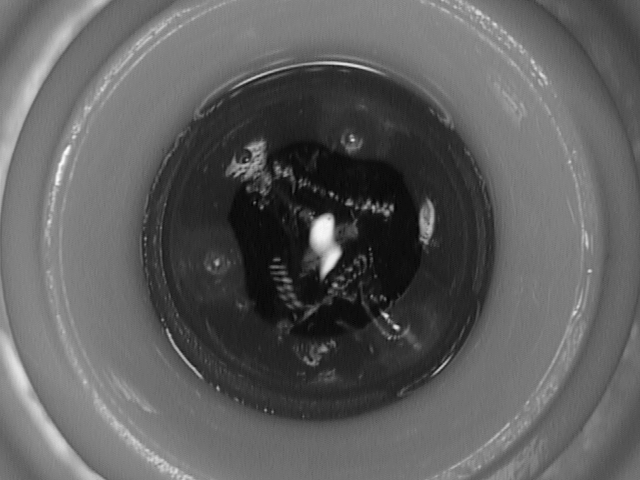 |
| 20 | BD | Q-Syte | 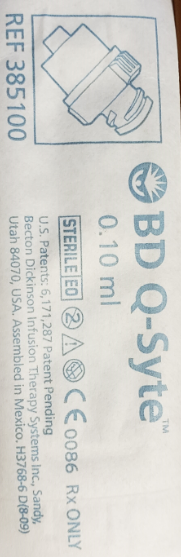 | Split Septum | 0.3419  0.3731  0.3586 | 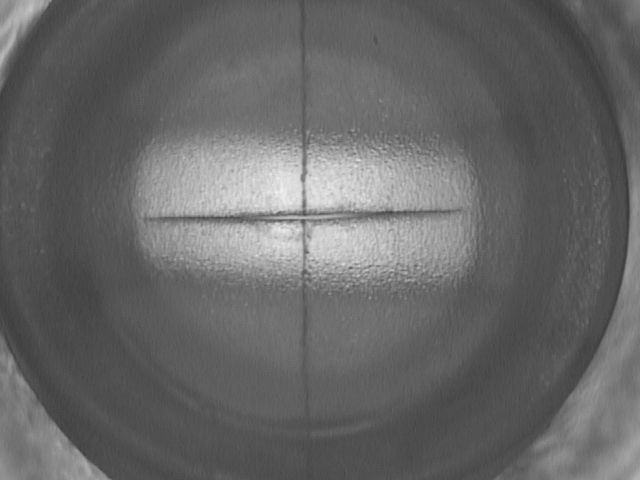 |

Note: Properties of flow path, access portal, and anti-reflux valve were apparent upon examination of each connector, while fluid displacement was reported by the manufacturer. Hydrodynamics were informed through prior work and visual analysis of the fluid path behavior for connectors.^8^ The seal length was measured using a Smartscope MVP (Optical Gaging Products, Rochester, NY) and fitting either a circle or line to the seal for each connector (n=3). Seal length (perimeter) was then calculated from the measured diameter, or the length reported for slit type connectors. Values represent the mean seal length. The flow path surface area and volume were calculated by recreating a 3D model of each connector and using the software (SolidWorks) to calculate the surface area or volume in contact with the fluid.

*Seal length was measured using TKO-6P instead of TKO-6. Of note, the slit on both connectors is the same, and the analysis was unchanged.

# **Supplemental Figures**

## **Figure S1.** Mean LDs per flush for each connector plotted across runs

Each point in the figure is the mean LD of bacteria in the flush, averaged across all inoculations, flushes, and replicate connectors within each run, and across all days in each run.

## **Figure S2.** Run mean biofilm LD attached to the connector plotted across runs

Each point in the above graph is the mean LD for a connector averaged across runs, days, and samples.

## **Figure S3.** Run mean biofilm LD attached to the hub plotted across runs

Each point in the above graph is the mean LD for a hub averaged across runs, days, and samples.

## **Figure S4.** Run mean biofilm LD attached to the segment plotted across runs

Each point in the above graph is the mean LD for a catheter segment averaged across runs, days, and samples.

## **Figure S5.** Run mean surface inoculation LDs (averaged across the two inoculations on each day and all days) plotted across runs

Each point in the graph is the mean LD (averaged across inoculations, days, and replicate connectors) of the surface controls for a single run. The solid line that connects the points shows how the surface controls trended over the runs.

**References**

1. Cullinane C. Right Management and Flushing. In: Moureau NL, editor. Vessel Health and Preservation: The Right Approach for Vascular Access. Cham: Springer International Publishing; 2019. p. 243-61.

2. Saefken B, Ruegamer D. Conditional Akaike information criterion for lme4, R package version 0.3. arXiv; 2018.

3. Saefken B, Ruegamer D, Kneib T, Greven S. Conditional Model Selection in Mixed-Effects Models with cAIC4. arXiv; 2018.

4. Nelson MT, LaBudde RA, Tomasino SF, Pines RM. Comparison of 3M Petrifilm Aerobic Count Plates to standard plating methodology for use with AOAC antimicrobial efficacy methods 955.14, 955.15, 964.02, and 966.04 as an alternative enumeration procedure: collaborative study. J AOAC Int. 2013;96(4):717-22.

5. Parker AE, Walker DK, Goeres DM, Allan N, Olson ME, Omar A. Ruggedness and reproducibility of the MBEC biofilm disinfectant efficacy test. J Microbiol Methods. 2014;102:55-64.

6. Fritz BG, Walker DK, Goveia DE, Parker AE, Goeres DM. Evaluation of Petrifilm™ aerobic count plates as an equivalent alternative to drop plating on R2A agar plates in a biofilm disinfectant efficacy test. Curr Microbiol. 2015;70(3):450-6.

7. FDA. Federal Register. Healthcare Antiseptics Final Rule. 2017;82:60487.

8. Nybo E, Maneval JE, Codd SL, Ryder MA, James GA, Woodbury J, et al. Flow velocity maps measured by nuclear magnetic resonance in medical intravenous catheter needleless connectors. J Pharm Biomed Anal. 2018;152:1-11.
